# Quantifying the impact of inhalational burns: a prospective study

**DOI:** 10.1186/s41038-018-0126-z

**Published:** 2018-09-04

**Authors:** Si Jack Chong, Yee Onn Kok, Rosanna Xiang Ying Tay, Desai Suneel Ramesh, Kok Chai Tan, Bien Keem Tan

**Affiliations:** 1 Department of Plastic, Reconstructive and Aesthetic Surgery, Singapore General Hospital, Level 5, Academia, Singapore, 169865 Singapore; 2 Department of Anaesthesiology, Singapore General Hospital, Level 5, Academia, Singapore, 169865 Singapore

**Keywords:** Inhalational, Burns, Injury, Acute kidney injury, Acute respiratory distress syndrome, Pneumonia

## Abstract

**Background:**

Inhalational injury is a major cause of morbidity and mortality in burns patients. This study aims to analyse the clinical outcomes, complications and bacteriology of inhalational burn patients.

**Methods:**

A prospective study was done on consecutive admissions to Burn Department, Singapore General Hospital over 15 months from January 2015 to March 2016. Presence of inhalational injury, demographics, complications and outcomes was recorded. Diagnosis of inhalational injury was based on history, symptoms and nasoendoscopy. Diagnosis of acute respiratory distress syndrome (ARDS), acute kidney injury (AKI) and infective complications were according to the Berlin criteria, acute kidney injury network (AKIN) classification stage 2 and above and the American Burns Association guidelines.

**Results:**

Thirty-five patients (17.3%) had inhalational burns out of 202 patients (63.4% male, 57.4% Chinese population). The average age was 43 ± 16.7 years (range 16–86), and percentage of total body surface area (%TBSA) was 12.1 ± 18.0 (range 0–88). In patients with inhalational injury, age was 38.9 ± 17.2 years and %TBSA was 30.3 ± 32.3. In patients without inhalational injury, age was 44.1 ± 12.8  years and %TBSA was 8.3 ± 9.59. Compared to patients with cutaneous injury alone, patients with inhalational burns had more surgeries (3 ± 7.07 vs 1 ± 1.54, *p* = 0.003), increased length of stay (21 days vs 8 days, *p* = 0.004) and higher in-hospital mortality rate (17.1% vs 0.6%, *p* < 0.001). Incidence of ARDS and AKI was 48.6% and 37.1%, respectively, compared to 0.6% and 1.2% in the patients without inhalational injury (*p* < 0.001). Patients with inhalational injury had increased incidence of bacteraemia (31.4% vs 2.4%, *p* < 0.001), pneumonia (37.1% vs 1.2%, *p* < 0.001) and burn wound infection (51.4% vs 25.1%, *p* = 0.004). Inhalational injury predicted AKI with an adjusted odds ratio (OR) of 17.43 (95% confidence interval (CI) 3.07–98.87, *p* < 0.001); ARDS, OR = 106.71 (95% CI 12.73–894.53, *p* < 0.001) and pneumonia, OR = 13.87 (95% CI 2.32–82.94, *p* = 0.004). *Acinetobacter baumannii* was the most frequently cultured bacteria in sputum, blood and tissue cultures with inhalational injury. Gram-negative bacteria were predominantly cultured from tissue in patients with inhalational injury, whereas gram-positive bacteria were predominantly cultured from tissue in patients without inhalational injury.

**Conclusions:**

Inhalational injury accompanying burns significantly increases the length of stay, mortality and complications including AKI, ARDS, infection and sepsis.

## Background

The importance of inhalational injury as a predictor of mortality is widely recognised [1–4]. Few prospective studies, especially in a tropical burns setting, compared clinical outcomes in patients with inhalational burns and those with cutaneous burns only [5]. This study aims to analyse the effect of inhalational injury on inpatient mortality, length of stay and complications.

## Methods

**Fig. 1:**
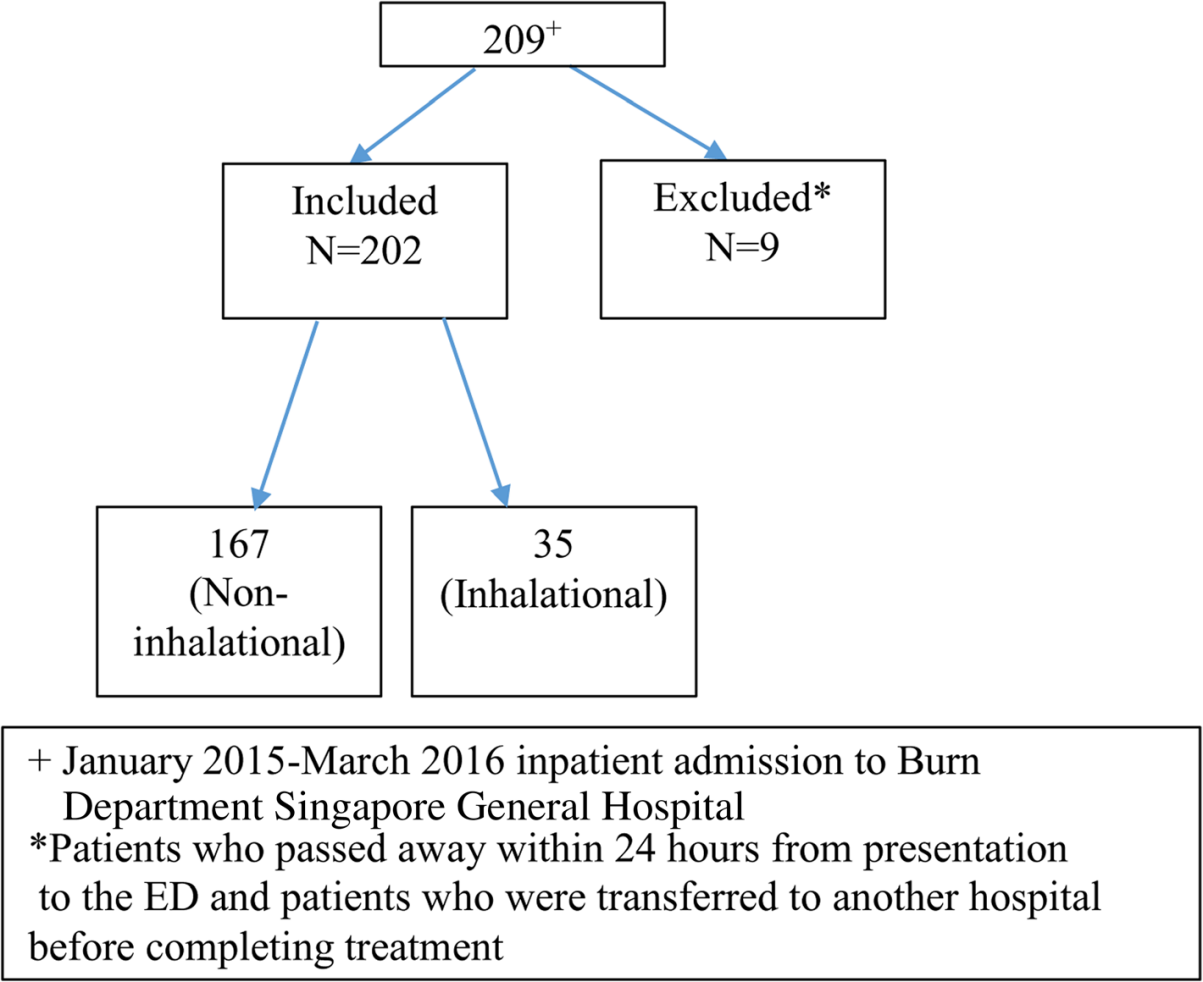
Patient recruitment flowchat

A prospective observational study was done on consecutive admissions to Burn Department, Singapore General Hospital from January 2015 to March 2016. Patients were excluded if they are deceased within 24 h of admission or transferred (Fig. 1). Data was collected on our Research Electronic Data Capture (REDCap) database. The study was approved by the Singapore General Hospital Institutional Review Board.

In-hospital mortality, number of surgeries, length of stay and percentage of total body surface area (%TBSA) were recorded. Complications analysed were acute kidney injury (AKI), acute respiratory distress syndrome (ARDS), pneumonia and culture-positive burns wound infection and bacteraemia.

### Statistical analysis

Data was analysed using IBM SPSS Statistics with institutional statistician’s help. *χ*^2^ and Mann-Whitney *U* tests were used. Binary logistic regression was applied to determine if inhalational injury was an independent predictor of complications, removing effect of %TBSA and age. *p* < 0.05 was considered to indicate a statistically significant result. Dependent variables were the outcome/complication, e.g., AKI. Covariates were inhalational injury, age and %TBSA.

### Inhalational injury

Inhalational injury was diagnosed by attending doctors in patients with history of smoke/fumes inhalation/fires in an enclosed space and three or more symptoms or signs and one positive finding on nasoendoscopy (Table 1).

**Table 1:** Symptoms and signs suggestive of inhalational injury

Symptoms and signs of inhalational injury	Nasoendoscopy
• Hoarseness of voice • Sore throat • Dyspnea • Painful respirations • Carbonaceous sputum • Tachypnea • Stridor • Facial burns/mucosal burns of lips and mouth • Singed facial hair/nasal hair • Soot in nostrils or mouth	• Airway erythema/edema • Soot in airways • Abnormal mobility and appearance of vocal cords

Bronchoscopy was performed only in patients with mucus plugging and was not part of diagnostic criteria. Patients referred from overseas with prior diagnosis were included.

#### Treatment principles

Patients were treated according to Advanced Burn Life Support (ABLS) [6] principles and modified Parkland’s regime (2–3 ml/kg/TBSA). In particular, if there was inhalational injury based on clinical findings or nasoendoscopy, patients with inhalational burns were monitored with hourly oxygen saturations and given a heparinised saline nebuliser (50 U heparin in 5 ml normal saline). Patients were intubated if findings on nasoendoscopy suggested severe airway injury with airway or vocal cord edema and if they had stridor or had an inappropriate PaO2 /FiO2 (P/F) ratio. Other factors taken into account include age—patients more than 60 years old with risk factors for poor cough reflex were intubated.

Ventilation was interchanged between conventional ventilation and airway pressure release ventilation (APRV) depending on patient response. Tidal volume was 6–8 ml/kg and positive end expiratory pressure (PEEP) was titrated to P/F ratio as in the ARDSNet study/protocol [7]. Prone positioning and neuromuscular paralysis are weak recommendations in the Surviving Sepsis Guidelines [8] and also not practical (in cases of facial swelling and abdominal distention). In refractory hypoxaemia, APRV is used as recruitment and haemodynamics are superior [9–11] with improved compliance, reduced duration of ventilation and lower mortality.

Indications for antibiotic therapy was for prophylaxis for all major burns > 20%, infected burns and 5 days post graft or biobrane. Intravenous cefazolin is the usual first line.

Surgical treatment strategy was for early biobrane (epidermal skin dressing) or excision within 48 h. Biobrane as the first surgery has dramatically reduced the need for subsequent excision in partial thickness burns by reducing burns conversion and providing ‘coverage’. Subsequent surgeries were for further excision and grafting if burns conversions, infection or biobrane non-take occurred.

### Non-infective complications

Berlin definition for ARDS was used [12]. Arterial blood gas was obtained in patients with inhalational injury, respiratory distress or abnormal chest radiographs. AKI was defined as ≥ acute kidney injury network (AKIN) classification stage 2 [13].

### Infective complications

The American Burns Association recommendations for diagnosis of infectious complications in patients with thermal trauma were followed [14]. In patients with clinical suspicion of pneumonia/infection, appropriate investigations were ordered and bacteria cultures were recorded.

Sputum cultures in patients with pneumonia were obtained via induced sputum with nebulised saline. Bronchoalveolar lavage (BAL) in patients with bronchoscopy was done in patients with desaturations refractory to mechanical ventilation.

## Results

Characteristics of patients included in this study were listed in Table 2. The most common mechanisms of injury were fire and scalds. Thirty-five (17.3%) patients had inhalational burns out of 202 patients (63.4% male, 57.4% Chinese population). The average age was 43 ± 16.7 years (range 16–86), and %TBSA was 12.1 ± 18.0 (range 0–88). In patients with inhalational injury, age was 38.9 ± 17.2 years and %TBSA was 30.3 ± 32.3. In patients without inhalational injury, age was 44.1 ± 12.8 years and %TBSA was 8.3 ± 9.59.

**Table 2:** Patient characteristics included in the study

Demographics	All patients *n* (%)	Patients with inhalational injury *n* (%)	Patients without inhalational injury *n* (%)	*p* value, *U*
Number	202 (100)	35 (17.3)	167 (82.7)	
Age (years)				
Mean (SD)	43.1 (16.7)	38.8 (17.25)	44.1 (12.78)	
Range	16–86	18–69	16–86	
Median (IQR)	42 (29–56)	43(29–58)	35 (30–48)	0.176, *U* = 2451
%TBSA, median (IQR)	5 (2.38–13.0)	5 (2.5–12)	9 (0–66)	0.036, *U* = 3582
Facial burns	72 (35.6)	18 (51.4)	54 (32.3)	0.032
Upper body burns	162 (80.2)	21 (60.0)	141 (84.5)	0.001
Sex				
Male	128 (63.4)	26 (74.2)	102 (61.1)	
Female	74 (36.6)	9 (25.7)	65 (38.9)	
Race				
Chinese	116 (57.4)	15 (42.8)	101(60.5)	
Indian	19 (9.4)	3 (8.6)	16 (9.6)	
Malay	22 (10.9)	4 (11.4)	18 (10.8)	
Others	45 (22.2)	13 (37.1)	32 (19.2)	

*SD* standard deviation, *IQR* interquartile range, *%TBSA* percentage of total body surface area

Compared to patients with cutaneous injury alone, patients with inhalational burns had more surgeries (3 ± 7.07 vs 1 ± 1.54, *p* = 0.003), increased length of stay (21 days vs 8 days, *p* = 0.004) and higher in-hospital mortality rate (17.1% vs 0.6%, *p* < 0.001) (Table 3).

**Table 3:** Clinical outcomes, complications and mortality of the burn patients

	Non-inhalational burns *n* = 167	Inhalational burns *n* = 35	*p* value, *U*
Number of surgery sessions, median (IQR)	1 (1–1)	3 (0–10)	0.003, *U* = 3552
Length of stay (days), median (IQR)	8 (5–12)	21 (4–43)	0.004, *U* = 3582
Average length of stay/%TBSA (sum of length of stay/sum of %TBSA)	1.307	0.976	NA
Length of ICU stay (days), median (IQR)	0 (0–0)	3 (0–10)	< 0.001, *U* = 4785
Average length of ICU stay/%TBSA (sum of length of ICU stay/sum of %TBSA)	0.020	0.313	NA
Days on IV antibiotics, median (IQR)	3 (1–6)	9.5 (0–28.25)	0.014, *U* = 3552
Average days on IV antibiotics/%TBSA (Sum of days of IV antibiotics/sum of %TBSA)	0.580	0.474	NA
Days of mechanical ventilation, median (IQR)	0 (0–0)	2 (0–10)	< 0.001
Initial P/F ratio, median (IQR)	532.6 (316.8–678.9)	370.4 (291.8–554.2)	0.191
Lowest P/F ratio in 2 days from admission, median (IQR)	518.5 (372.0–689.5)	253.6 (202.7–408.4)	0.026
Lowest P/F ratio in 7 days from admission, median (IQR)	518.5 (372.0–689.5)	246.8 (181.5–389.4)	0.018
Admission to HD, *n* (%)	46 (27.5)	31(88.6)	< 0.001
Admission to ICU, *n* (%)	10 (6.0)	22 (62.9)	< 0.001
Intubation, *n* (%)	4 (2.4)	22 (62.8)	< 0.001
Mortality, *n* (%)	1 (0.6)	6 (17.1)	< 0.001
AKI, *n* (%)	2 (1.2)	13 (37.1)	< 0.001
ARDS, *n* (%)	1 (0.6)	17 (48.6)	< 0.001
Bacteraemia, *n* (%)	4 (2.4)	11 (31.4)	< 0.001
Pneumonia, *n* (%)	2 (1.2)	13 (37.1)	< 0.001
Burn wound infection, *n* (%)	42 (25.1)	18 (51.4)	0.004

*%TBSA* percentage of total body surface area, *ARDS* acute respiratory distress syndrome, *AKI* acute kidney injury, *IV* intravenous, *ICU* intensive care unit, *NA* not applicable, *HD* high dependency unit, *IQR* interquartile range, *P/F* PaO2 /FiO2

Incidence of ARDS and AKI  was 48.6% and 37.1%, respectively, compared to 0.6% and 1.2% in the patients without inhalational injury (*p* < 0.001). Patients with inhalational injury had increased incidence of bacteraemia (31.4% vs 2.4%, *p* < 0.001), pneumonia (37.1% vs 1.2%, *p* < 0.001) and burn wound infection (51.4% vs 25.1%, *p* = 0.004). Inhalational injury predicted AKI with an adjusted odds ratio (OR) of 17.43 (95% confidence interval (CI) 3.07–98.87, *p* < 0.001); ARDS, OR of 106.71 (95% CI 12.73–894.53, *p* < 0.001) and pneumonia, OR of 13.87 (%95 CI 2.32–82.94, *p* = 0.004) (Table 4).

**Table 4:** Adjusted odds ratios (OR) of getting poorer clinical outcomes compared to the median, adjusted for age and %TBSA (except age—adjusted for %TBSA, %TBSA—adjusted for age only)

	Non-inhalational burns *n* (%)	Inhalational burns *n* (%)	*p* value	Adjusted OR*	95% CI for OR	*p* value
%TBSA > median,5	92 (55.1%)	24 (68.6%)	0.142	1.66	0.76–3.64	0.204
Age > median, 42 (years)	88 (52.7%)	14 (40.0%)	0.172	0.71	0.31–1.62	0.414
Number of surgery sessions > median, 1	131 (78.4%)	23 (65.7%)	0.108	0.21	0.07–0.62	0.004
Length of stay > median, 8.5 (days)	79 (47.3%)	22 (62.9%)	0.094	1.12	0.47–2.67	0.797
Days on IV antibiotics > median, 4	83 (49.7%)	22 (62.9%)	0.157	0.86	0.36–2.11	0.749
Days of mechanical ventilation > median, 2.5	3 (1.8%)	15 (42.9%)	< 0.001	18.38	2.97–113.68	0.002
Initial P/F ratio < median, 394.02	3 (1.8%)	14 (40.0%)	< 0.001	44.85	9.10–221.13	< 0.001
Lowest P/F ratio < median, 343.88	1 (0.6%)	15 (42.9%)	< 0.001	140.58	13.14–1504.25	< 0.001
Lowest P/F ratio < 300	1 (0.6%)	17 (48.6%)	< 0.001	480.70	28.47–8115.89	< 0.001
Mortality	1 (0.6%)	6 (17.1%) P/F **<** 300:5/17(29.4%) P/F **>** 300:1/18 (5.6%)	< 0.001	0.604	0.01–44.53	0.818

*Odds ratio adjusted for %TBSA and age

*%TBSA* percentage of total body surface area, *CI* confidence interval, *P/F* PaO2 /FiO2, *IV* intravenous

Patients with inhalational burns had a higher %TBSA. Seventeen out of 35 patients fitted the Berlin criteria (P/F ratio < 300 and within 7 days). This is reflected in Table 4 (lowest P/F ratio < 300). Within the group of PIB, those with P/F ratios < 300 in the first week had a higher in-hospital mortality (29.4%) compared to those with P/F ratios > 300 in the first week (5.56%) (*p* = 0.08). We could not get statistical significance when adjusted for %TBSA and for both age and %TBSA (Table 5).

**Table 5:** Adjusted odds ratios of complications in patients with inhalational injury using logistic regression analysis

	Inhalational injury			Inhalational injury, adjusted for %TBSA		Inhalational injury, adjusted for %TBSA and age (except %TBSA, adjusted for age only)			
	OR	95% CI	*p* value	Adjusted OR	95% CI	*p* value	Adjusted OR	95% CI	*p* value
%TBSA > median, 5	1.78	0.82–3.87	0.146	–	–	–	1.66	0.76–3.64	0.204
Age > median, 42 (years)	0.60	0.29–1.26	0.175	0.71	0.31–1.62	0.414	–	–	–
All complications	8.33	3.62–19.17	< 0.001	5.31	2.17–12.98	< 0.001	6.27	2.49–15.77	< 0.001
Intubation	68.96	20.65–230.3	< 0.001	67.19	12.47–361.89	< 0.001	86.99	14.22–544.43	< 0.001
Pneumonia	48.75	10.30–230.55	< 0.001	13.87	2.32–82.94	0.004	35.51	3.90–323.07	0.002
ARDS	156.78	19.69–1248.17	< 0.001	106.71	12.73–894.53	< 0.001	480.70	28.47–8115.89	< 0.001
AKI	48.75	10.31–230.55	< 0.001	17.43	3.07–98.87	< 0.001	31.71	4.38–229.7	0.001
Bacteraemia	18.68	5.50–63.39	< 0.001	2.39	0.38–15.04	NS	2.56	0.40–16.57	NS
Burn wound infection	3.15	1.49–6.67	0.003	1.28	0.49–3.35	NS	1.35	0.52–3.54	NS
Mortality	34.35	3.99–295.86	0.001	0.38	0.01–11.29	NS	0.60	0.01–44.53	NS
HD admission	20.39	6.82–60.95	< 0.001	15.70	4.87–50.62	< 0.001	15.55	4.82–50.20	< 0.001
ICU admission	26.57	10.41–67.90	< 0.001	16.26	5.23–50.57	< 0.001	16.63	5.28–52.34	< 0.001
Number of surgery sessions > median, 1	0.53	0.24–1.16	0.111	0.22	0.08–0.64	0.005	0.21	0.07–0.62	0.004
Length of stay > median, 8.5	1.89	0.89–3.99	0.098	1.05	0.44–2.47	0.915	1.12	0.47–2.67	0.797
Days on IV antibiotics > median, 4	1.71	0.81–3.63	0.160	0.84	0.35–2.04	0.699	0.86	0.36–2.11	0.749
Days of mechanical ventilation > median 2.5	41.00	10.91–154.04	< 0.001	11.94	2.34–60.8	0.003	18.38	2.97–113.682	0.002
Initial P/F ratio < median 394.02	36.45	9.67–137.40	< 0.001	28.89	7.02–118.89	< 0.001	44.85	9.10–221.13	< 0.001
Lowest P/F ratio in 2 days < median, 343.88	124.50	15.61–993.29	< 0.001	78.88	9.28–670.76	< 0.001	140.58	13.14–1504.25	< 0.001
Lowest P/F ratio in 1 week < 300	156.78	19.69–1248.17	< 0.001	106.72	12.73–894.54	< 0.001	480.70	28.47–8115.89	< 0.001

*OR* odds ratio*, CI* confidence interval, *NS* non-significant, *ARDS* acute respiratory distress syndrome, *AKI* acute kidney injury, *IV* intravenous, *RF* respiratory failure, *ICU* intensive care unit, *HD* high dependency unit, *%TBSA* percentage of total body surface area, *SD* standard deviation, *CI* confidence interval, *P/F* PaO2 /FiO2

### Bacteriology

**Fig. 2:**
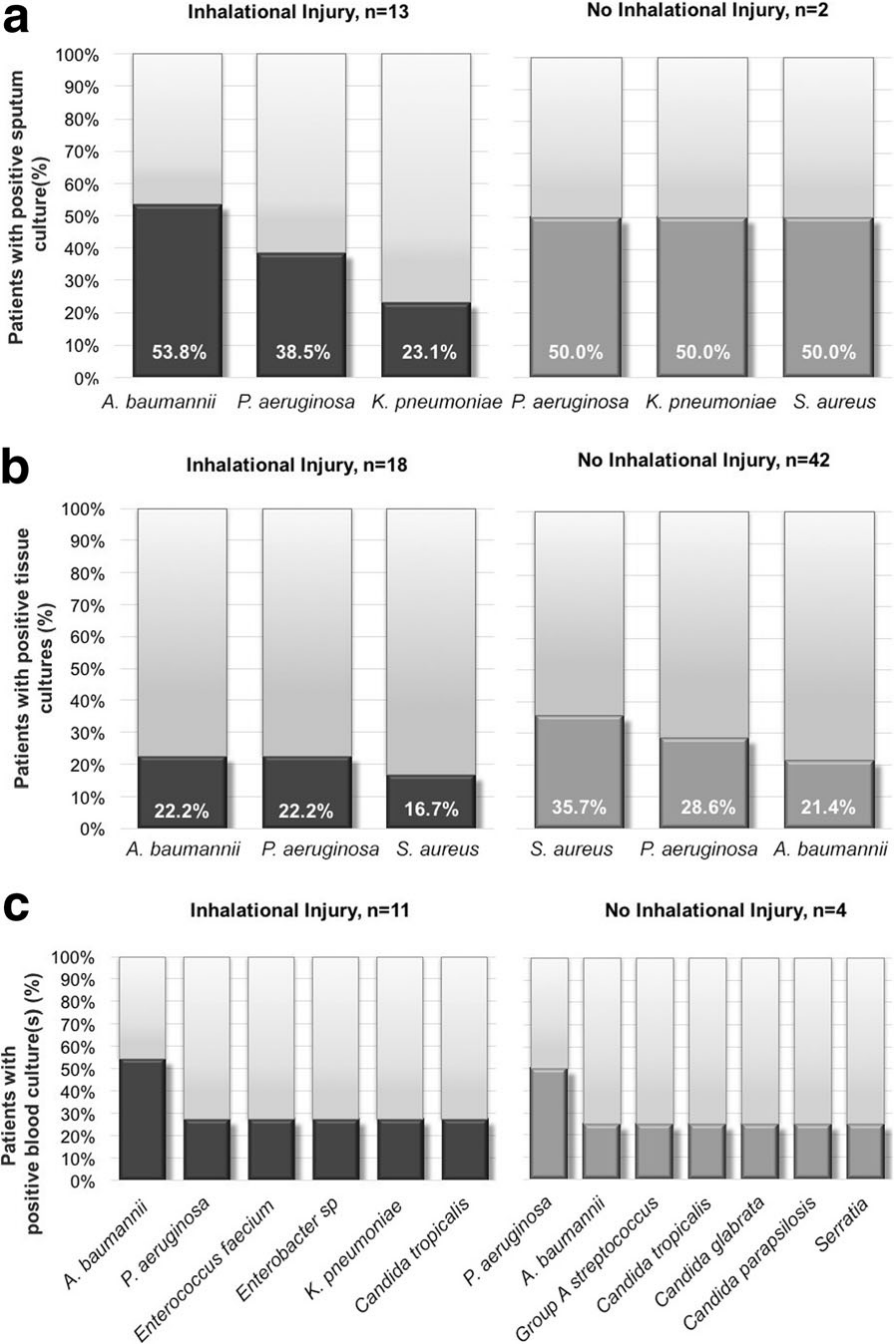
Chart showing most common bacteria grown in sputum (**a**), tissue (**b**) and blood (**c**) cultures in patients with inhalational burns and patients with cutaneous burns only

In patients with infective complications, cultures were taken and the number of positive cultures for a microbe was expressed as a percentage of all positive cultures (Fig. 2).

In patients with inhalational injury, *Acinetobacter baumannii* (*A. baumannii*) was the most frequently cultured in sputum, tissue and blood cultures.

Pneumonia was most frequently caused by *A. baumannii* followed by *Pseudomonas aeruginosa (P. aeruginosa)* and *Klebsiella pneumoniae (K. pneumoniae)*.

These three species were also the most common in the patients with bacteraemia together with *Candida tropicalis*, *Enterobacter* and *Enterococcus faecium*. In tissue cultures, *A. baumannii* and *P. aeruginosa* were most common followed by *Staphylococcus aureus (S. aureus).*

*A. baumannii* was less common in patients without inhalational injury.

In patients without inhalational injury, burn wound infections were most commonly due to *S. aureus* followed by *P. aeruginosa* and *A. baumannii*. In those with inhalational injury, *A. baumannii* and *P. aeruginosa* were more common.

## Discussion

Burn management has improved over the past decades with advances in fluid resuscitation, nutritional support, pulmonary care and infection control practices. This includes improvements in wound care/dressing, such as our previously described enhanced total body wrap technique using negative pressure wound therapy, which protects the wound from micro-organisms and provides an efficient channel to clear excessive exudate while keeping the wounds moist [15]. This reduces frequency of dressing changes and lowers infection rates through prevention of strikethrough [15]. Of note, incidence of burn wound infections in our centre have been reducing over the years, from 50.2% in a 2003–2005 study [5] to 29.7% in our study. Currently, infectious and respiratory complications remain a leading cause of morbidity. In burns cases with %TBSA of 40–75%, almost all deaths are due to sepsis from infectious complications and/or inhalation injury [16].

It is well-reported that inhalational injury results in longer stays and higher mortality which results in significant economic burden. In the current study, we prospectively followed patients in a burn unit and quantified the impact of inhalational injury on clinical outcomes and complications. All patients were managed uniformly using our institution’s burns protocol.

### Mortality

Our study found that presence of inhalational injury was associated with increased mortality. The overall in-hospital mortality was 3.5%. In-hospital mortality in patients with inhalational injury was 17.1%, compared to those without inhalational injury of 0.6%.

The benchmark inhalational mortality is high and ranges from 9.5 to 46.6% internationally. Typically, patients with respiratory failure from inhalational burns have up to 50% risk of mortality. Our mortality rate of 17.1% (6 patients) was likely due to concomitant respiratory failure, large %TBSA burns (> 40%) and accompanying renal failure, accounting for their high mortality rate. The 6 patients were 23–48 years old, typically with severe Endorf-Gameli type 3 or 4 bronchoscopy findings with delayed resuscitation and debridement (72 h). The crux in further reduction of inhalational mortality we believe lies in early aggressive burns resuscitation, coverage and aggressive intensive care unit (ICU) respiratory care to modulate the systemic inflammatory response syndrome (SIRS) inflammatory response.

Statistical significance was not reached for mortality when adjusting for %TBSA, possibly due to the low mortality in the group without inhalational injury.

Inhalational injury has been shown to be a significant predictor of mortality in various multivariable retrospective studies on the prognostic factors in burn patients (Table 6).

**Table 6:** Retrospective studies on inhalational injury, mortality, length of stay and other clinical outcomes

	Our study SGH Chong	Texas Shirani 1987 [17]	Utah Hollingsed 1993 [18]	North Carolina Smith 1994 [19]	Tokyo Suzuki 2005 [2]	Egypt El-Helbawy 2011 [4]	Taiwan Chen 2014 [1]	USA Anand 2015 [3]
*n*	202	1058	529	1447	5560	281	21,791	506,628
Type of study	Prospective study	Retrospective single centre	Retrospective single centre	Retrospective single centre	Retrospective multicentre	Retrospective single centre	Retrospective multicentre	Retrospective multicentre
PIB (%)	17.3	35	5.7	19.6	30.4	46.3	7.9	3.47
Age (years)	(median) Overall: 42 PIB: 43 PCB: 35	PCB: 27 Abnormal xenography: 37 Abnormal bronchoscope: 39	(mean) PIB: 20.2 RF: 36.6	(mean) Overall: 30 (3 months–93 years)	(mean (SD)) Overall: 40.1 (26.2) PIB: 49.0 (20.5) PCB: 36.2(27.4)	–	(mean(SD)) Overall: 30.9 (22.6)	(mean) Overall: 30
%TBSA	(median) Overall: 5 PIB: 5 PCB: 9	PCB: 23 Abnormal xenography: 37 Abnormal bronchoscope:50	(mean) PIB: 16.2 RF: 40.4	(mean)18% (0.4–100%)	mean (SD)) Overall: PIB: 29.9 (30.3) PCB: 16.1 (17.3)	–	mean Overall: 12.2%	–
Mortality (%)	Overall 3.5 PIB: 17.1 PCB: 0.6	Overall: 22.7 PIB: 46.6 PCB: 9.6	Overall: 6.2 PIB -without RF:0 - with RF: 27 PCB: - without RF: 3 - with RF: 50	Overall 9.5 PIB 31 PCB 4.3	Overall: 15.8 PIB: 33.6 PCB: 8.1	Overall: 23.1 PIB: 41.5 PCB: 7.2	Overall 2.1 PIB: 17.9 PCB: 0.76	Overall 3.73 PIB: 4× increase in mortality vs PCB
Length of stay (days)	PIB: 21 PCB: 8	–	PIB - without RF: 17.8 - with RF: 42.6 PCB - without RF: 15.4 -with RF: 43.2	–	–	–	–	PIB: 9 PCB: 6
Other outcomes	Pneumonia ARDS, AKI infection IV antibiotics, ICU days	Pneumonia PIB: 38% PCB: 8.8%	ARDS PIB: 20% PCB: 2%	–	–	–	Rate of dying due to pneumonia, sepsis and wound infection is higher in PIB	Hospital charges (median) PIB: US$32,070 PCB: US$ 17,600

*PIB* patients with inhalational burns, *PCB* patients with only cutaneous burns, *ARDS* acute respiratory distress syndrome, *AKI* acute kidney injury, *IV* intravenous, *RF* respiratory failure, *ICU* intensive care unit, *%TBSA* percentage of total body surface area, *SD* standard deviation

Shirani et al. estimated that the burn-related death rate is 20% higher in patients with combined inhalation injury and cutaneous burns than in those with cutaneous burns alone [17]. In a 2014 Taiwanese study, 21,791 burns patients from 44 hospitals were retrospectively reviewed. The overall mortality rate was 2.1%, and inhalation injuries were found in 7.9% of the patients. The mortality rate of inhalation and non-inhalation injury group was 17.9% and 0.7%, respectively, similar to our results [1]. In Suzuki et al.’s 17-year study, the mortality rate was 15.8% and multivariable analysis revealed inhalational injury to be the most important predictor of death [2]. In Smith et al's study, inhalational injury was a significant predictor for mortality, but the most important predictor of mortality was still %TBSA [18].

As in Table 6, the mortality ranges from 17% to 46.6% in inhalational burns. The reasons for high mortality in inhalational burns despite low %TBSA cutaneous burns is because of associated pneumonia, SIRS, sepsis and associated organ failure [1–3, 17].

### ARDS

Patients with inhalational injury had > 100 times increase in developing ARDS. Incidence of ARDS in patients with inhalational injury was 48.6%, compared to 0.6% in the other group. A 4-year review of 529 burns patients reported a 20% incidence of ARDS, compared to 2% in patients without inhalational injury (*p* < 0.001) [19].

During inhalational injury, the inflammatory response forms airway casts and cause V/Q mismatch, pulmonary edema and cell death, promoting airway occlusion and pulmonary dysfunction [1]. In patients with large cutaneous burns, capillary hyper-permeability occurs not only at the injured site, but also in regions distant from the injury [20, 21] resulting in worsening of pulmonary edema. This systemic response is more severe when the thermal injury is associated with smoke inhalation [22].

### AKI

Inhalational injury was also a predictor of AKI. In Coca’s retrospective study, inhalational injury was an independent predictor of AKI (OR = 3.6 (95% CI 1.76–7.34), *p* < 0.001) [23]. Other significant factors in multivariable analysis were catheter infections and sepsis. Both inhalational injury and AKI were independent predictors of mortality.

### Pneumonia

In this study, inhalational injury was an independent risk factor for pneumonia. It was associated with 13 times higher likelihood of pneumonia, independent of %TBSA. Shirani et al.’s hallmark study quantitated that both inhalational injury and pneumonia have contributions to mortality that are independent and additive [17]. In that study, expected mortality increased by a maximum of 20% in the presence of inhalation injury alone, 40% in the presence of pneumonia alone and 60% when both inhalation injury and pneumonia were present.

### Length of stay

After adjusting for %TBSA (Table 3), ICU stay and length of stay was longer for inhalational burns due to respiratory complications. Days of intravenous (IV) antibiotics were comparable. This is a result of more operative sessions, complications and longer duration of IV antibiotics. We postulate the increase in operative sessions to be due to increased wound infections requiring debridement and wound conversion due to systemic sepsis, cytokine alteration and inflammatory response. A study using the National Inpatient Sample database of 506,628 admissions found inhalational injury was a factor for length of stay (OR = 2.01 (95% CI 1.84–2.21), *p* < 0.001) [3].

### Bacteriology

Patients with inhalational burns were infected with gram-negative organisms, common agents for invasive infection. The prevalence of *A. baumanni* in our tropical burns unit has been previously reported [5, 24]**.** *A. baumanni* is endemic to the hospital because of the tropical climates and the particular high survivability of Acinetobacter even in adverse conditions [24] The most common agents were *A. baumanni* followed by methicillin-resistant *Staphylococcus aureus* (MRSA) then *P. aeruginosa* [24]. Interestingly, *A. baumann*i was the most common aetiologic agent in our patients with inhalational burns, but not in patients without inhalational burns.

Patients with burns are exposed to infections from loss of the skin barrier. In patients with inhalational burns, the respiratory tract is also damaged. The longer length of stay in hospital and duration of IV antibiotics may account for prevalence of resistant gram-negative nosocomial infections in inhalational burns patients. In an Indian study, gram-positive organisms were predominantly cultured during the first week of admission whereas gram-negative organisms with increasing levels of resistance were common from the second week onwards [16]. In patients with major burns, we may occasionally find multi-resistance organisms developing at 1–2 weeks of stay, which may be caused by antibiotic use.

### Limitations

The generalizability of our results may be limited by the lack of bronchoscopy as an objective criterion for inhalational injury and different grades of severity. Our criterion for inhalational injury was a clinical diagnosis without taking into account bronchoscopy—a widely used technique [25–28] for diagnosing inhalational injury based on presence of hyperaemia, edema and soot [25]. A clinical criterion is readily applied in triage for quick stratification into patients with inhalational injury at high risks of the complications analysed in our paper. However, this diagnoses inhalational injury as being either present/absent instead of providing a scale of injury severity based on bronchoscopy proven lower tract injury. There is a lack of standard consensus in various clinical criteria, making inter-centre data comparisons difficult. Another limitation is that of inter-observer bias and reliability in each of the clinical criteria. We have optimised the variability by ensuring final diagnostic assessments are by attendings.

Bronchoscopy is an objective means of diagnosing and grading inhalational injury—with several grading systems proposed. This can potentially allow inter-centre comparisons should a standardised and validated grading system be accepted [25]. However, if bronchoscopy were to be done for diagnostic purposes in all patients in our study, some patients would be subjected to a bronchoscopy not otherwise indicated for diagnostic/therapeutic purposes. In patients with unlikely lower airway injury, it may not be necessary to pass the endoscope below the true vocal folds for negative diagnosis.

Bronchoscopy may be uncomfortable for the patient and requires careful anaesthesia of the larynx to avoid obstruction due to laryngospasm in a potentially inflamed airway. Furthermore, some institutions may not have the equipment or expertise for bronchoscopy. Fiberoptic laryngoscopy is better tolerated by the patients, requires minimal medication, and is often more readily available [26]. One study has addressed this with an objective decision tree that can be readily replicated in different centres, including bi-level endoscopic examination of the oro-rhino-pharynx and tracheobronchial tree and also clinical criteria [25]. Studies have discussed using bronchoscopy to evaluate severity of inhalational burns [27, 28]. Several scales of severity have been proposed; these have not been able to predict clinical outcomes [25, 27].

Further bacteriology studies can include analysis of time from injury to the date of culture for bacteriology and resistance of bacteria cultured.

## Conclusion

Inhalational injury is associated with increased length of stay, morbidity and complications such as ARDS, AKI, pneumonia and infection. Mortality is higher (29.4% to 5.6%) if P/F is < 300. Counselling patients and their family regarding the associated morbidity is essential. Statistical significance was not reached when adjusted for %TBSA and age. Larger sample sizes are needed. Inhalational burn care is an important step towards advancing burn management, given its association with morbidity. Bronchoscopy classification is also encouraged as this was lacking in this study. This requires early recognition of inhalational burns, early lung recruitment protocol, early intubation, early ICU care, earlier lavage and earlier burns debridement to reduce the SIRS response.

## Abbreviations

AKI: Acute kidney injury
APRV: Airway pressure release ventilation
ARDS: Acute respiratory distress syndrome
CI: Confidence interval
ICU: Intensive care unit
IV: Intravenous
MRSA: Methicillin-resistant *Staphylococcus aureus*
NS: Not significant
OR: Odds ratio
PCB: Patients with only cutaneous burns
PEEP: Peak end expiratory pressure
PIB: Patients with inhalational burns
REDCap: Research Electronic Data Capture
RF: Respiratory failure
%TBSA: Percentage of total body surface area
